# The intricate role of Sir2 in oxidative stress response during the post-diauxic phase in *Saccharomyces cerevisiae*

**DOI:** 10.3389/fmicb.2023.1285559

**Published:** 2023-11-09

**Authors:** Yeong Hyeock Kim, Ji-In Ryu, Mayur Nimbadas Devare, Juhye Jung, Jeong-Yoon Kim

**Affiliations:** Department of Microbiology and Molecular Biology, College of Bioscience and Biotechnology, Chungnam National University, Daejeon, Republic of Korea

**Keywords:** *Saccharomyces cerevisiae*, Sir2, oxidative stress, Ras2, cytosolic pH, Azf1

## Abstract

Silent information regulator 2 (Sir2) is a conserved NAD^+^-dependent histone deacetylase crucial for regulating cellular stress response and the aging process in *Saccharomyces cerevisiae*. In this study, we investigated the molecular mechanism underlying how the absence of Sir2 can lead to altered stress susceptibilities in *S. cerevisiae* under different environmental and physiological conditions. In a glucose-complex medium, the *sir2*Δ strain showed increased sensitivity to H_2_O_2_ compared to the wild-type strain during the post-diauxic phase. In contrast, it displayed increased resistance during the exponential growth phase. Transcriptome analysis of yeast cells in the post-diauxic phase indicated that the *sir2*Δ mutant expressed several oxidative defense genes at lower levels than the wild-type, potentially accounting for its increased susceptibility to H_2_O_2_. Interestingly, however, the *sir2*Δ*ras2*Δ double mutant exhibited greater resistance to H_2_O_2_ than the *ras2*Δ single mutant counterpart. We found that the expression regulation of the cytoplasmic catalase encoded by *CTT1* was critical for the increased resistance to H_2_O_2_ in the *sir2*Δ*ras2*Δ strain. The expression of the *CTT1* gene was influenced by the combined effect of *RAS2* deletion and the transcription factor Azf1, whose level was modulated by Sir2. These findings provide insights into the importance of understanding the intricate interactions among various factors contributing to cellular stress response.

## Introduction

Oxidative stress is caused by an imbalance between the production of reactive oxygen species (ROS) and the capacity for oxidative stress resistance. ROS, such as superoxide radicals, hydrogen peroxide, and hydroxyl radicals are generated during normal cellular metabolism in addition to ATP generation in mitochondria (Finkel and Holbrook, 2000). While ROS can be beneficial in cell signaling and host defense in small amounts, excessive levels can cause oxidative damage to nucleic acids, proteins, and lipids (Finkel and Holbrook, 2000; Bartosz, 2009; Barry and Gutteridge, 2015). Over time, cumulative damage can contribute to aging and a wide range of diseases, including neurodegenerative disorders, cardiovascular disease, and cancer (Finkel and Holbrook, 2000; Schieber and Chandel, 2014). To counteract the harmful impact of ROS, cells have evolved intricate antioxidant defenses, which consist of enzymes like superoxide dismutase, catalase, and glutathione peroxidase, as well as small molecule antioxidants such as vitamins C and E (Finkel and Holbrook, 2000; de la Torre-Ruiz et al., 2015). The coordinated cellular processes activated in response to oxidative stress include the upregulation of antioxidant defenses, repair of damaged molecules, and removal of damaged proteins and organelles (Barry and Gutteridge, 2015; de la Torre-Ruiz et al., 2015).

*Saccharomyces cerevisiae* has been widely used as a model organism in oxidative stress research. When subjected to oxidative stress, *S. cerevisiae* activates a complex network of mechanisms involving MAPK pathways, such as Hog1, Slt2, and Fus3/Kss1, which respond dynamically to a range of stress conditions (Morano et al., 2012; de la Torre-Ruiz et al., 2015). *S. cerevisiae* modulates the activities of key transcription factors that control the expression of antioxidant enzymes. These enzymes serve as the first line of defense against oxidative stress by neutralizing ROS and mitigating oxidative damage. Transcription factors such as Yap1, Skn7, and Msn2/4 are crucial in *S. cerevisiae*’s response to oxidative stress (He and Fassler, 2005; Morano et al., 2012). Yap1 is crucial for regulating the expression of several antioxidant enzymes, such as catalase and superoxide dismutase. Skn7 cooperates with Yap1 in regulating some oxidative stress genes, while Msn2/4 is responsible for the general stress response (Hasan et al., 2002; He and Fassler, 2005; de la Torre-Ruiz et al., 2015). This highly coordinated response underscores *S. cerevisiae*’s ability to adapt to changing environmental conditions and stresses.

Silent information regulator (Sir2), a conserved NAD-dependent histone deacetylase, is a well-established regulator of aging in various organisms, including yeast, flies, and mammals (Tissenbaum and Guarente, 2001; Rogina and Helfand, 2004; Satoh et al., 2013). Apart from its role in extending replicative lifespan (RLS) by preventing the formation of extrachromosomal rDNA circles (Kaeberlein et al., 1999) and deacetylating histone H4 lysine 16 (H4K16) at subtelomeric regions (Dang et al., 2009), Sir2 has been shown to reduce ROS levels and the accumulation of oxidative damage in daughter cells (Aguilaniu et al., 2003; Erjavec et al., 2007). Additionally, Sir2 has been observed to modulate the transcription of antioxidant genes in a growth phase-dependent manner (Kang et al., 2014). However, the precise molecular mechanisms by which Sir2 participates in oxidative stress response still need to be fully understood due to the complex influence of other signaling pathways and genetic factors.

The Ras/cAMP/PKA signaling pathway is widely recognized as a crucial component in the cellular response to oxidative stress (Fabrizio et al., 2005; Creamer et al., 2022). Strains with a deletion of *RAS2* have been shown to exhibit increased resistance to H_2_O_2_ and extended chronological lifespan, pointing to a negative correlation between Ras2 activity and oxidative stress/longevity (Fabrizio et al., 2001; Mirisola and Longo, 2022). Although these findings underscore the significance of the Ras/cAMP/PKA pathway in regulating oxidative stress, the potential involvement of the sirtuin protein, Sir2, in Ras2’s regulatory mechanisms has largely remained unexplored. Our study illuminates the molecular mechanisms underlying the varied role of Sir2 in oxidative stress regulation during the post-diauxic phase. Specifically, we demonstrate that Ras2, whose activity is regulated by pH, determines Sir2’s role in H_2_O_2_ resistance by regulating *CTT1* gene expression during the post-diauxic phase.

## Materials and methods

### Yeast strains and media

Unless otherwise stated, all the experiments were performed using DBY746 (*MATα leu2-3*, *112 his3D trp1-289 ura3-52 GAL*^+^) cells. Cells were grown in a standard liquid YPD medium containing yeast extract (10 g/L, Becton Dickinson), peptone (20 g/L, Becton Dickinson), and glucose (20 g/L, Junsei), with pH adjusted to 6.0 for all experiments. A synthetic drop-out medium was prepared by adding 0.67 g/L yeast nitrogen base without amino acids (Becton Dickinson) and amino acids, except uracil or histidine, to select transformant cells. When required, transformant cells were plated onto a solid medium containing 5′-fluoroorotic acid (1 mg/mL) to select for the loss of the *URA3* marker.

### Stress resistance test

Unless otherwise stated, all stress resistance tests were conducted on cells in the post-diauxic phase, grown in YPD medium with a pH of 6.0. For the H_2_O_2_ resistance assay, cells were diluted to an OD_600_ of 1 in 0.1 M potassium phosphate buffer (pH 6.0) and treated with the appropriate concentration of H_2_O_2_ for 30 min. Heat stress resistance assays were performed by diluting cells to an OD_600_ of 1 in distilled water and incubating them at 55°C (heat-shocked) or 30°C (control) for 60–120 min. SDC medium was used for the H_2_O_2_ resistance assay, prepared by adding yeast nitrogen base without amino acids (0.67 g/L), glucose (20 g/L), and supplemented with amino acids as well as a 4-fold excess of leucine, histidine, tryptophan, and uracil. After stress exposure, cells were serially diluted, spotted onto YPD plates, and incubated at 30°C for 2–3 days.

### Western blotting

Cell extracts were prepared using the trichloroacetic acid (TCA) method, and the pellet was resuspended in a sample loading buffer. For Ahp1, cells were lysed in lysis buffer (50 mM HEPES, 140 mM NaCl, 1 mM EDTA, 1% Triton X-100, 1 mM PMSF). Supernatants were collected after centrifugation and resuspended in a sample loading buffer. Protein samples were separated on 8%–12% SDS-polyacrylamide gel electrophoresis gels and transferred to polyvinylidene fluoride membrane (Millipore, Billerica). The membrane was probed with specific antibodies and detected using HRP-conjugated secondary antibodies. Primary antibodies used were anti-Flag (1:2000; Sigma), anti-GFP (1:1000; Santa Cruz Biotechnology), and anti-GAPDH (1:20000; Acris). Band density was quantified using ImageJ software (National Institutes of Health).

### Measurement of the cytosolic pH_c_

The cytosolic pH was measured following a previously described method (Devare et al., 2020). To generate pH calibration curves, yeast cells expressing SEP (kindly provided by Daniel E. Gottschling) under the control of the *TEF1* promoter were cultivated in baffled flasks until an OD_600_ of approximately 1.0 in YPD medium. Subsequently, the cells were centrifuged at 3,000 rpm for 5 min, washed twice with PBS, and resuspended in PBS supplemented with 5 μg/mL digitonin (Sigma). After an incubation period of 5 min, the cells were washed again with PBS and resuspended in citric acid/Na_2_HPO_4_ buffer with pH values ranging from 5.5 to 8.0. The cytosolic pH values of viable single cells during the exponential, post-diauxic, or buffered post-diauxic growth phase were determined. Imaging was carried out using an Olympus BX51 microscope, and ImageJ software was utilized for the analysis. Mean pHluorin intensity was quantified from three different regions of images using a 1-pixel straight-line tool. The pH values were always presented as mean ± SD. The pH determination experiments were performed three times (biological repeats), and the figures show one representative experimental result, where the error bars represent the standard deviation of at least three replicates.

### Measurement of Ras2 activity

To measure Ras2 activity, cells expressing EGFP-3x RBD were grown in YPD medium with or without citrate phosphate buffer and harvested at either the exponential phase (6 h) or the post-diauxic phase (24 h). The localization of EGFP-3x RBD was analyzed using an Olympus BX51 microscope. SC-URA medium with or without glucose was used as a control to test whether the localization of EGFP-3x RBD was affected by the presence of glucose.

### Chronological lifespan

The chronological lifespan of cells incubated in YPD medium was monitored by measuring colony-forming units (CFUs) every 2–3 days. The number of CFUs on day 3 was considered the initial survival (100%) and was used to determine the age-dependent mortality.

### RNA isolation, cDNA synthesis, and real-time PCR analysis

Total RNA was isolated using the NucleoSpin RNA kit (Macherey-Nagel) and quantified by measuring absorbance at 260 nm. From 1 μg of RNA sample, cDNA was synthesized using the ReverTra Ace qPCR RT kit (Toyobo) according to the manufacturer’s recommendations and analyzed by quantitative RT-PCR. RT-PCR was performed using SYBR green PCR mix and CFX connect system (Bio-Rad). The relative expression levels normalized to ACT1 were determined using the comparative CT method. For RNA sequencing analysis, post-diauxic phase DBY746 and *sir2∆* cells at 6 h for the exponential phase or 24 h for the post-diauxic phase were harvested, and total RNA was isolated and purified as described above before being sent for sequencing.

### Immunoprecipitation

Post-diauxic phase cells were harvested, washed one time with cold distilled water, and resuspended in the lysis buffer containing 50 mM HEPES, 140 mM NaCl, 1 mM EDTA, 1% Triton X-100, 1 mM PMSF, 10 mM NaF and 2 mM Na_3_VO_4_. The lysate was collected by centrifugation, and the supernatant was immunoprecipitated using anti-Flag or anti-Sir2 antibody (Santa Cruz Biotechnology) pre-conjugated with protein A/G beads overnight. The immunoprecipitates were washed five times with lysis buffer and then eluted by boiling in sample loading buffer. The protein samples were separated by 8% SDS-PAGE and transferred onto PVDF membrane for western blotting analysis using appropriate antibodies.

## Results

### The role of Sir2 in oxidative stress response varies depending on culture condition

**Figure 1:**
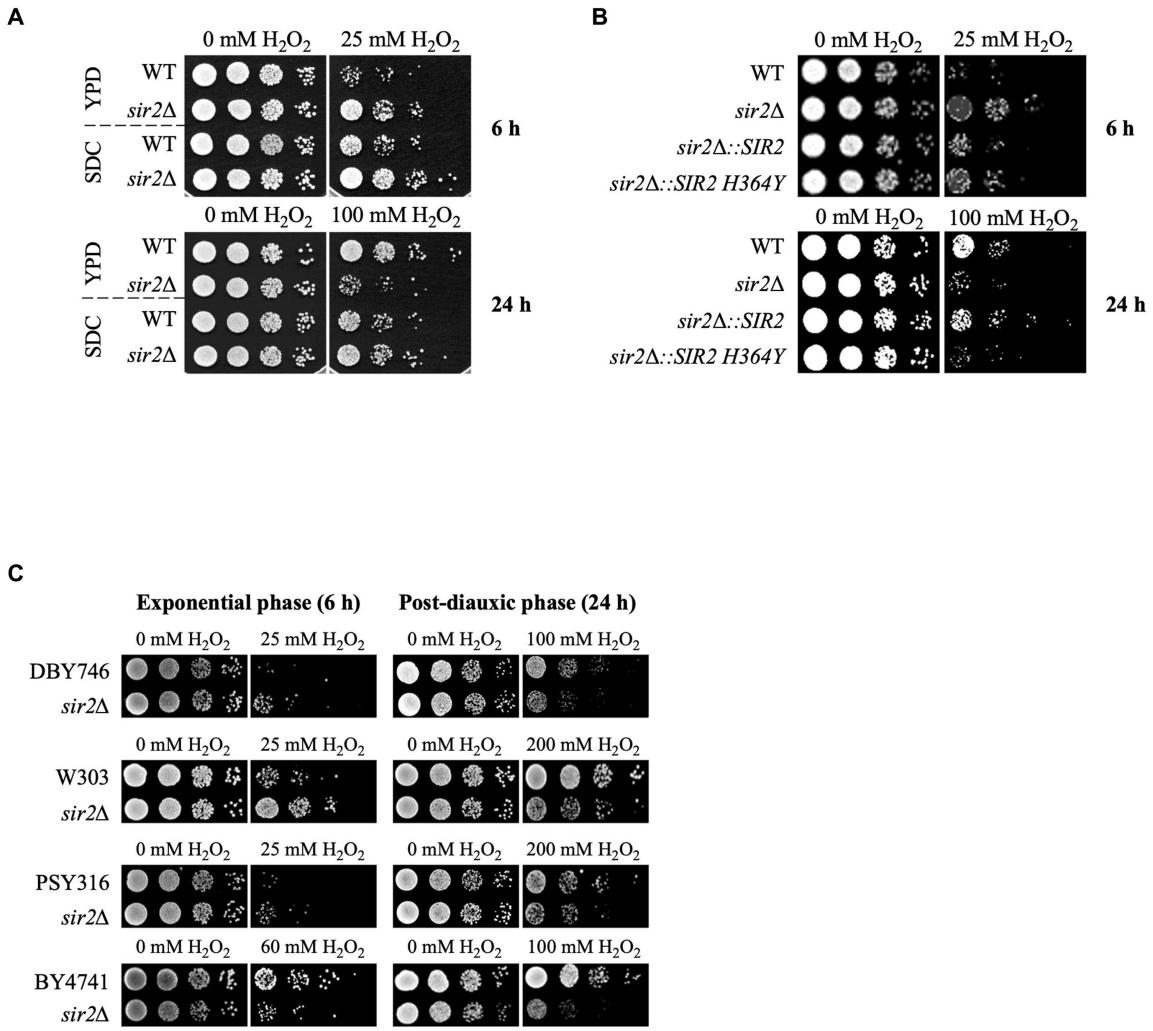
Context-dependent role of Sir2 in H_2_O_2_ resistance. **(A)** H_2_O_2_ resistance was assessed in the wild-type (DBY746) and *sir2*∆ mutant in SDC and YPD media during the exponential or post-diauxic phase was tested. **(B)** H_2_O_2_ resistance was measured in DBY746, *sir2*∆, and *sir2*∆ carrying either the wild-type *SIR2* or enzymatically inactive *SIR2 H364Y* allele in the exponential and post-diauxic growth phases. **(C)** H_2_O_2_ resistance was tested in DBY746, W303, PSY316, and BY4741 strains and their respective *sir2*∆ mutants in the exponential and post-diauxic growth phases. Cells were treated with the indicated amount of H_2_O_2_ for 30 min in potassium phosphate buffer (pH 6.0) at 30°C, spotted onto YPD plates, and incubated at 30°C for 2–3 days.

In a previous study, we reported that Sir2 has positive and negative roles in oxidative stress response and lifespan extension (Kang et al., 2014). To further understand the underlying mechanisms, we investigated the oxidative stress sensitivity of the *sir2*Δ strain under two different culture conditions: the glucose-complex YPD medium and glucose-minimal SDC medium. In the YPD medium, the *sir2*Δ strain exhibited greater sensitivity to H_2_O_2_ than the wild-type during the post-diauxic phase, consistent with previous findings. Conversely, during the exponential growth phase, the *sir2*Δ strain showed increased resistance to H_2_O_2_ compared to the wild-type (Figure 1A). However, in the SDC medium, even during the post-diauxic phase, the *sir2*Δ strain was less sensitive to H_2_O_2_ than the wild-type (Figure 1A). Furthermore, cells expressing the enzymatically inactive variant of Sir2 (Sir2-H364Y) displayed a phenotype similar to that of the *sir2*Δ strain, indicating that the deacetylase activity of Sir2 is required for its sensitivity to H_2_O_2_ in the post-diauxic phase (Figure 1B). Additionally, during the post-diauxic phase in the YPD medium, we observed that *sir2*Δ mutants derived from various *S. cerevisiae* strains were more sensitive to H_2_O_2_ than their wild-type counterparts (Figure 1C), suggesting that the negative role of Sir2 in H_2_O_2_ sensitivity during the post-diauxic phase is generalizable across diverse *S. cerevisiae* strains.

### Sir2 affects the expression of oxidative stress resistance genes during the post-diauxic phase

**Figure 2:**
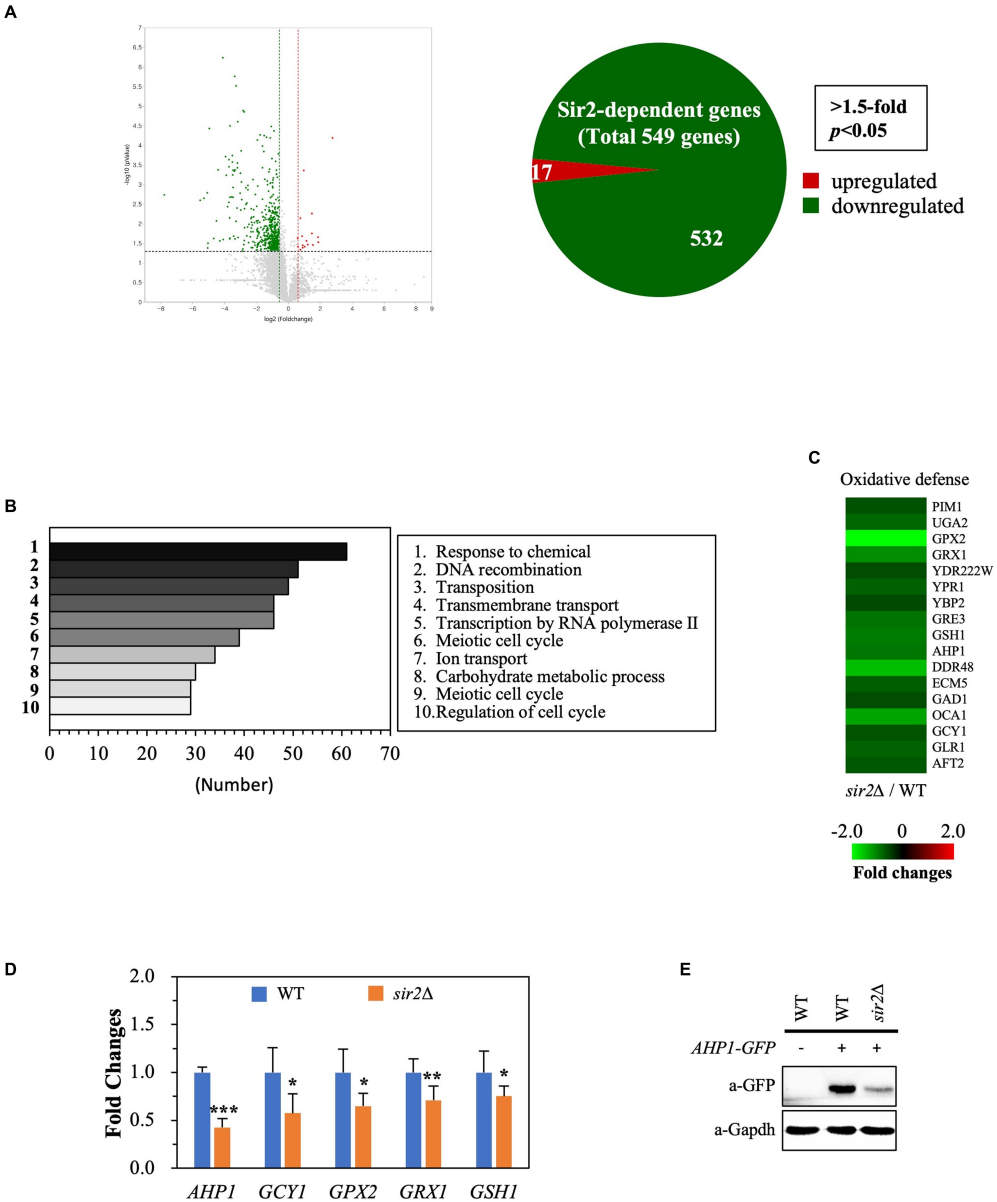
Sir2 regulates the expression of genes involved in oxidative stress resistance. Total RNA was extracted from DBY746 and *sir2∆* strains in the post-diauxic phase (24 h), subjected to RNA sequencing, and verified by qRT-PCR and western blot. **(A)** Volcano plot and Venn diagram show the number of genes upregulated or downregulated in the *sir2*∆ strain compared to the wild-type (DBY746) strain, with a fold change greater than 1.5 and *p* < 0.05. The RNA sequencing data set is available on figshare at https://doi.org/10.6084/m9.figshare.24055566.v1. **(B)** The top 10 categories identified by Gene Ontology (GO) analysis, representing genes downregulated in the *sir2*∆ strain compared to the wild-type strain, are presented. **(C)** Heat maps depict the relative mRNA levels of genes involved in oxidative stress, with fold changes greater than 1.5 and *p* < 0.05. **(D)** qRT-PCR analysis of selected genes related to oxidative stress resistance in the wild-type and *sir2∆* strains is presented. **(E)** Western blot analysis shows the Ahp1 protein levels in the wild-type and *sir2∆* strains. GAPDH was used as a loading control. The reported values are the average of at least three independent experiments (±SD), and *p*-values were calculated using a *t*-test (^*^p < 0.05, ^**^p < 0.01, and ^***^p < 0.005).

To understand why the role of Sir2 in oxidative stress resistance changes during the post-diauxic phase in the YPD complex medium, we conducted transcriptome analyses comparing gene expression patterns between the wild-type and *sir2*Δ strains during the post-diauxic phase in the YPD medium. We found that the absence of Sir2 affected the expression of 549 out of 6,692 genes analyzed (*p* < 0.05, 1.5-fold), with 17 genes upregulated and 532 genes downregulated in the *sir2*Δ strain compared to the wild-type (Figure 2A). Among the 17 upregulated genes, none were associated with oxidative stress resistance or other significant biological processes. Interestingly, many downregulated genes belonged to oxidative stress resistance and chaperone functions, in addition to the categories of “response to chemical,” “DNA recombination,” and “transcription by RNA polymerase II” (Figures 2B,C). We confirmed the decreased expression of oxidative stress resistance genes, such as *AHP1*, *GCY1*, *GPX2*, *GRX1*, and *GSH1*, by qRT-PCR (Figure 2D) and further demonstrated that the decreased mRNA level of the *AHP1* gene encoding thioredoxin peroxidase was reflected in the amount of the Ahp1 protein (Figure 2E). These data strongly suggest that the absence of Sir2 results in decreased expression of oxidative stress resistance genes during the post-diauxic phase in the YPD medium, consequently making yeast cells more susceptible to oxidative stress.

### Altered cytosolic pH affects Sir2’s role in H_2_O_2_ resistance during the post-diauxic phase

**Figure 3:**
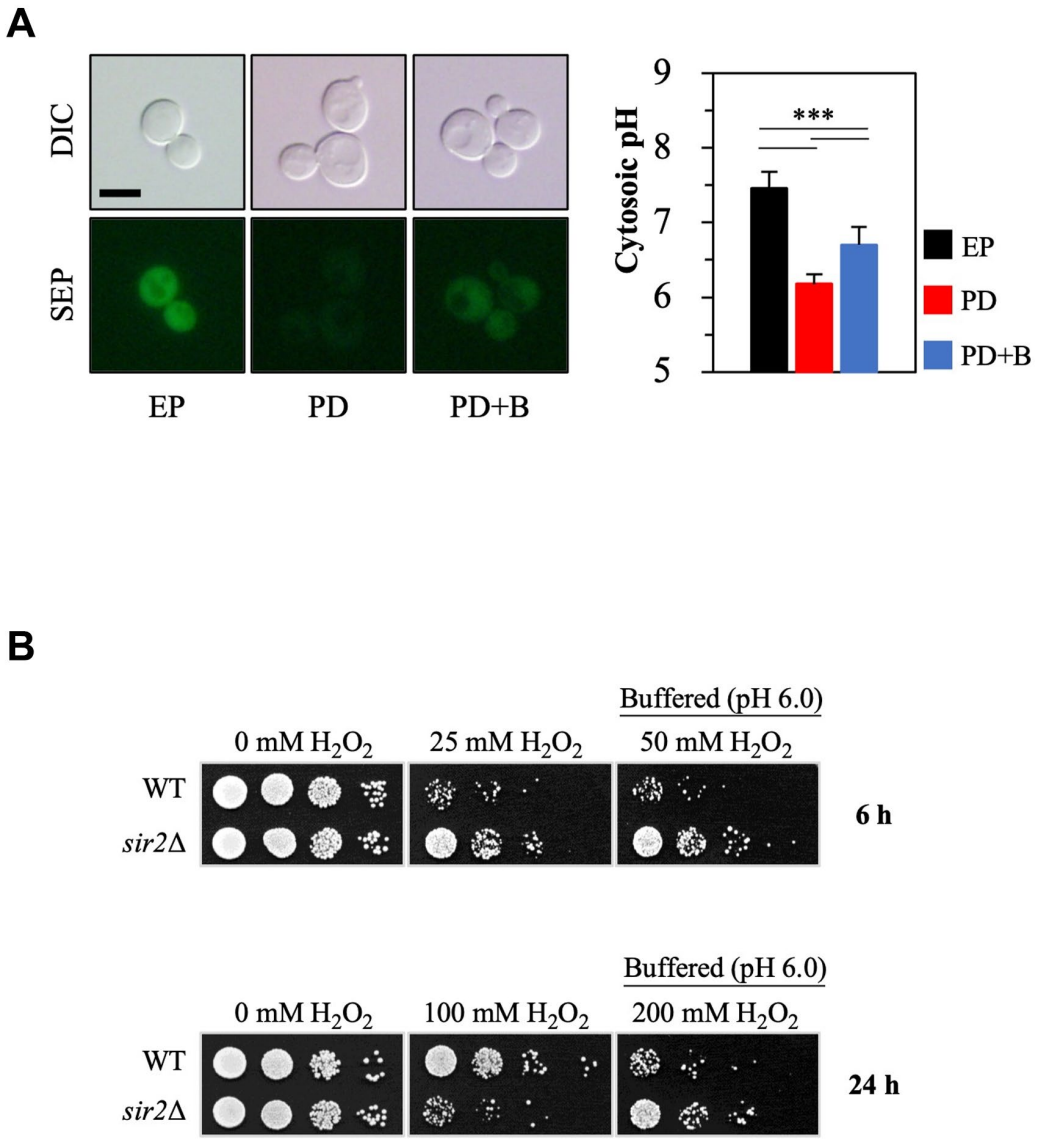
Increasing cytosolic pH by manipulating extracellular pH conditions changes Sir2’s role in H_2_O_2_ resistance during the post-diauxic phase. **(A)** Cytosolic pH was measured using cells expressing Super Ecliptic pHluorin (SEP) under the control of the *TEF1* promoter. Cells were prepared from the exponential phase (EP), post-diauxic phase (PD), and post-diauxic phase buffered with 0.1 M citrate phosphate buffer (pH 6.0, PD + B). DIC, differential interference contrast. The reported values are the average of at least three independent experiments (±SD), and *p*-values were calculated using a *t*-test (^***^p < 0.005). **(B)** H_2_O_2_ resistance was tested with the wild-type and *sir2*∆ cells growing under the indicated conditions.

Small cytosolic pH (pHc) changes can significantly affect cellular physiology (Orij et al., 2011). The pHc in yeast cells is not constant; it varies during growth and is influenced by external acidity levels. During the post-diauxic phase, the pHc is generally lower than in the exponential phase (Figure 3A), consistent with earlier findings (Dolz-Edo et al., 2019; Devare et al., 2020). To investigate whether this lowered pHc in the post-diauxic phase affects the sensitivity to oxidative stress, we increased the pHc by treating yeast cells in the post-diauxic phase with spent medium that contained 0.1 M citrate phosphate buffer (pH 6.0) (Figure 3A). Interestingly, the *sir2*Δ cells exposed to the buffered spent medium were more resistant to H_2_O_2_ than the control (Figure 3B), suggesting that a low pHc may contribute to the increased H_2_O_2_ sensitivity of the *sir2*Δ cells compared to the wild-type in the post-diauxic phase.

### Ras2 is responsible for different responses to H_2_O_2_ stress during the post-diauxic phase

**Figure 4:**
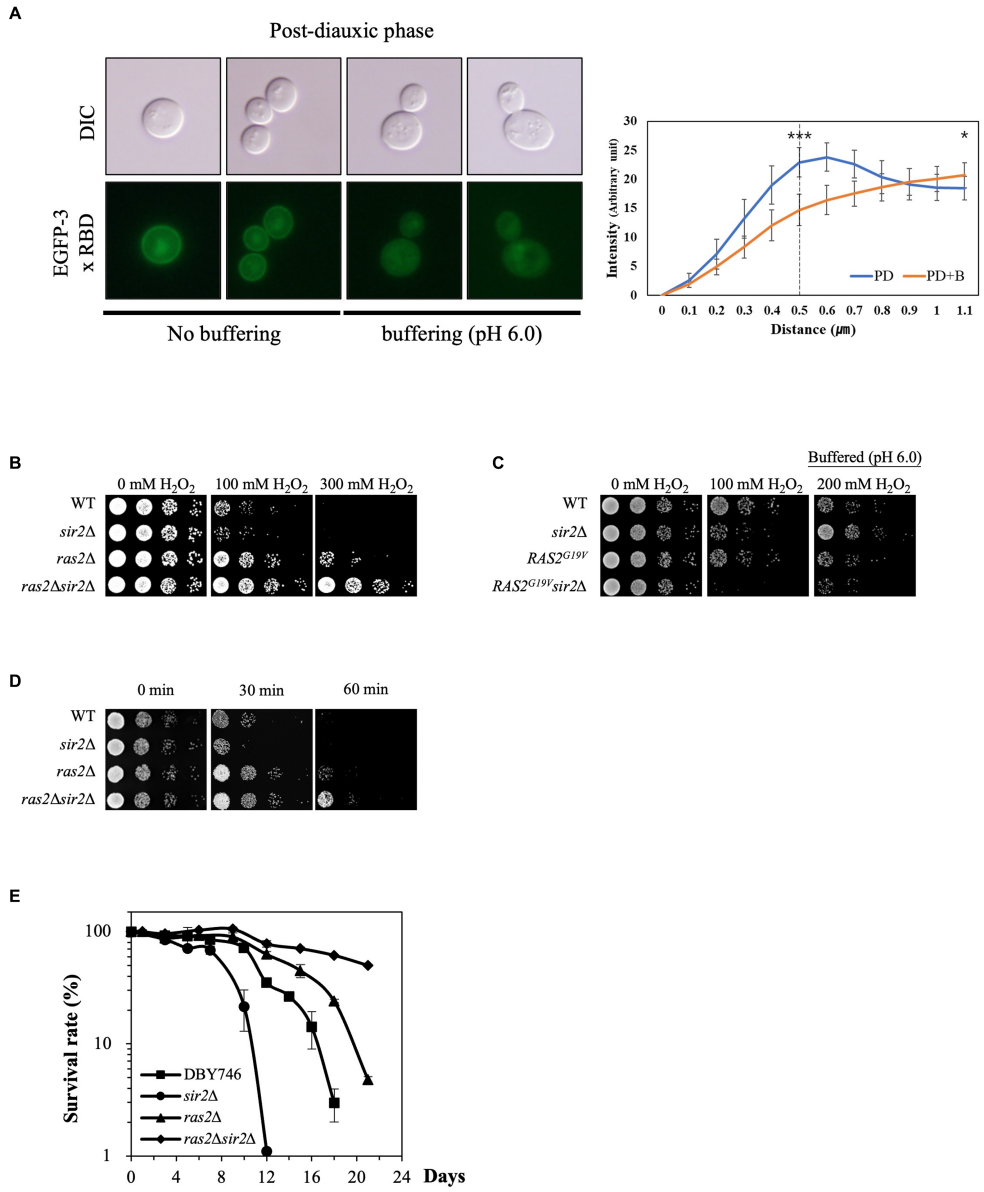
Ras2 activity is involved in the altered effect of *SIR2* deletion on H_2_O_2_ resistance during post-diauxic growth phases. **(A)** (Left panel) Ras2 activity was analyzed using EGFP-3x RBD in the wild-type strains treated with or without buffer (pH 6.0). (Right panel) The fluorescence intensity was assessed using ImageJ and expressed in arbitrary units (*n* = 10 cells for each strain). The distance is measured starting from a point outside the cell and traversing through the cell membrane. The dotted line at 0.5 μm serves as indicator of the approximate boundary between the interior and exterior of the membrane. *p*-values were calculated using a *t*-test (^*^p < 0.05 and ^***^p < 0.005). **(B)** H_2_O_2_ resistance was evaluated in the wild-type, *sir2*Δ, *ras2*Δ, and *ras2*Δ*sir2*Δ cells during the post-diauxic phase. **(C)** H_2_O_2_ resistance was tested in the wild-type, *sir2*Δ, *RAS2^G19V^*, and *RAS2^G19V^sir2*Δ strains during the post-diauxic phase. Note that *RAS2^G19V^* is a constitutively active form of *RAS2*. **(D)** Heat stress resistance was evaluated in the wild-type, *sir2*Δ, *ras2*Δ, and *ras2*Δ*sir2*Δ cells during the post-diauxic phase. **(E)** The chronological lifespan of the wild-type, *sir2*Δ, *ras2*Δ, and *ras2*Δ*sir2*Δ strains grown in YPD medium was monitored by counting colony-forming units every 2 or 3 days. Experiments were repeated three times. Error bars indicate the mean ± SD.

Since low pHc activates Ras2, a critical player in stress resistance, by inhibiting Ira1/2 (Tamanoi, 2011), we hypothesized that Ras2 might be involved in the altered sensitivity of the *sir2*Δ strain to oxidative stress during the post-diauxic phase. To visualize Ras2 activation, we fused the Ras binding domain (RBD) of human Raf1 with EGFP. In the presence of glucose, EGFP-RBD localized to the plasma membrane, indicating active Ras2. In contrast, in the absence of glucose, it dispersed into the cytoplasm, indicating inactive Ras2 (Supplementary Figure S1). During the post-diauxic phase, EGFP-RBD was localized to the plasma membrane. However, treatment with buffered medium displaced EGFP-RBD to the cytoplasm (Figure 4A), suggesting that buffering to pH 6.0 may result in Ras2 inactivation by increasing pHc.

To further investigate Ras2’s role in the greater H_2_O_2_ stress resistance of the *sir2*Δ cells than the wild-type during the post-diauxic phase, we deleted *RAS2* from the wild-type and *sir2*Δ mutant strains and assessed their H_2_O_2_ stress resistance. Remarkably, *RAS2* deletion reversed the effect of the *sir2* mutation on H_2_O_2_ stress resistance, similar to what was observed with buffered medium treatment. The *sir2*Δ *ras2*Δ double mutant displayed greater resistance to H_2_O_2_ than the *ras2*Δ single mutant (Figure 4B). In addition, we found that the expression of a constitutively active form of *RAS2* (*RAS2^G19V^*) increased the sensitivity of the *sir2*Δ cells to H_2_O_2_ stress (Figure 4C). Since oxidative stress is known to be involved in heat-induced cell death in yeast (Davidson et al., 1996), we compared the strains’ resistance to heat stress and found results consistent with those observed for H_2_O_2_ stress resistance (Figure 4D). These results collectively suggest that Ras2 activity is associated with the changes in the effects of *SIR2* deletion on oxidative stress resistance during different growth phases. However, the reason for the significant increase in stress resistance in the *sir2*Δ compared to the wild-type upon Ras2 inactivation remains unclear. Lastly, the chronological lifespan (CLS) of the strains was assessed. Consistent with previous results (Fabrizio et al., 2003), the *ras2*Δ strain exhibited an increased chronological lifespan, which was further enhanced in the *ras2*Δ*sir2*Δ strain (Figure 4E).

### *SIR2* deletion affects the expression of *CTT1* in the absence of Ras2, but not in the presence of Ras2, during the post-diauxic phase

**Figure 5:**
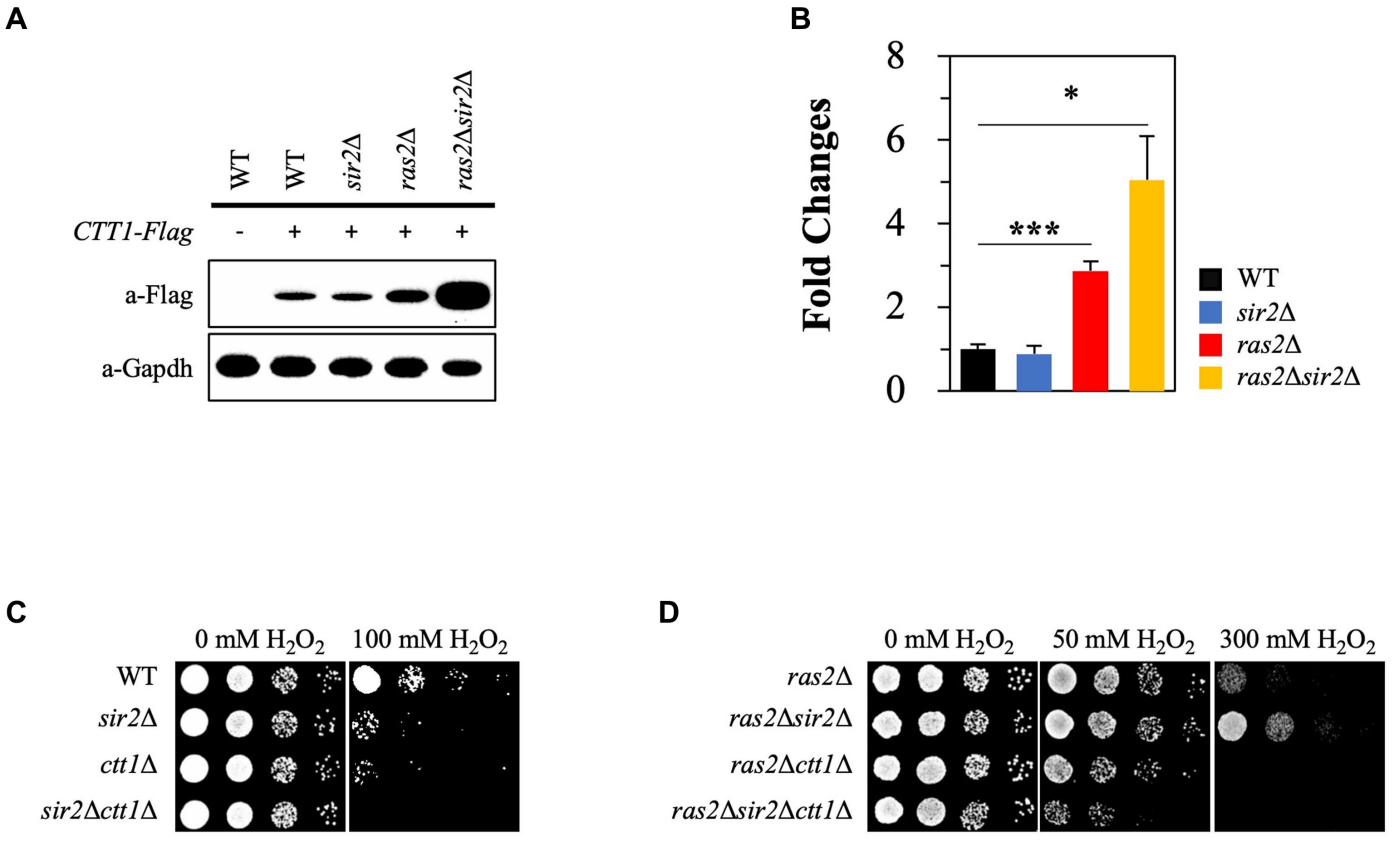
Sir2 affects the expression of *CTT1* in the absence of Ras2 but not in its presence. **(A)** Ctt1 protein levels in the wild-type, *sir2*Δ, *ras2*Δ, and *ras2*Δ*sir2*Δ strains were measured by western blot. GAPDH was used as a loading control. **(B)** qRT-PCR was performed to assess *CTT1* mRNA levels in the wild-type, *sir2*Δ, *ras2*Δ, and *ras2*Δ*sir2*Δ. The data represent the average of at least three independent experiments (±SD), and *p*-values were calculated using a *t*-test (^*^p < 0.05 and ^***^p < 0.005). **(C)** H_2_O_2_ resistance was tested in the wild-type, *sir2*∆, *ctt1∆*, and *sir2∆ctt1∆* strains. **(D)** H_2_O_2_ resistance was also tested in the *ras2*∆, *ras2*∆*sir2*∆, *ras2*∆*ctt1∆*, and *ras2*∆*sir2∆ctt1∆* strains during the post-diauxic phase.

Catalase is essential in oxidative stress resistance by breaking H_2_O_2_ into oxygen and water molecules. Previous studies suggested that the Ras2 signaling pathway negatively influences *CTT1* expression (Bissinger et al., 1989; Belazzi et al., 1991). Based on the studies, we hypothesized that intracellular acidification could activate Ras2 signaling during the post-diauxic phase, thus leading to decreased *CTT1* expression. To test this hypothesis, we analyzed *CTT1* expression in WT, *sir2*Δ, *ras2*Δ, and *sir2*Δ *ras2*Δ strains. As expected, we observed that *CTT1* expression was increased in the *ras2*Δ strain compared to the WT and *sir2*Δ strains. However, it was surprising that *CTT1* expression was significantly higher in the *sir2*Δ *ras2*Δ strain than in the *ras2*Δ strain (Figure 5A). The relative mRNA levels of the *CTT1* gene matched with the amounts of the Ctt1 protein in the strains (Figure 5B). To see whether the amount of the Ctt1 protein in the strains is related to the different oxidative stress resistance, we deleted the *CTT1* gene from WT, *sir2*Δ, *ras2*Δ, and *sir2*Δ *ras2*Δ strains and spotted them on plates after treating them with H_2_O_2_. We found that the resistance displayed by *ras2*Δ and *sir2*Δ *ras2*Δ strains was eliminated by *CTT1* deletion (Figures 5C,D). These results support our hypothesis that reduced pHc during the post-diauxic phase activates Ras2 signaling, which in turn suppresses *CTT1* expression and amplifies oxidative damage. These findings explain why Ras inactivation resulted in much higher stress resistance in the *sir2*Δ mutant than in the wild-type.

### Azf1 is involved in the regulation of *CTT1* expression

To investigate how *SIR2* deletion increases *CTT1* expression only in the absence of Ras2, we examined whether Msn2/4 transcription factors and Rim15 kinase play a role in the varied effects of *SIR2* deletion on *CTT1* expression, given their pivotal roles in linking Ras-PKA signaling to *CTT1* expression (Bissinger et al., 1989; Belazzi et al., 1991; Plank, 2022). We found that the deletion of *RIM15* has no effect on *CTT1* expression in the *ras2*Δ and *sir2*Δ*ras2*Δ strains (Supplementary Figure S2A). Deletion of *MSN2/4* significantly reduced *CTT1* expression in the *ras2*∆ and *ras2*∆*sir2*∆ mutants. However, *CTT1* expression was higher in the *ras2*∆*msn2*∆*msn4*∆*sir2*∆ strain than in the *ras2*∆*msn2*∆*msn4*∆ strain (Supplementary Figures S2B,C). Additionally, the *ras2*∆*msn2*∆*msn4*∆*sir2*∆ strain was slightly more resistant to H_2_O_2_ than the *ras2*∆*msn2*∆*msn4*∆ strain (Supplementary Figure S2D). These data indicate that while Msn2/4 are crucial for *CTT1* expression, they are not involved in the varied effect of *SIR2* deletion on *CTT1* expression in the *ras2*∆ strain.

**Figure 6:**
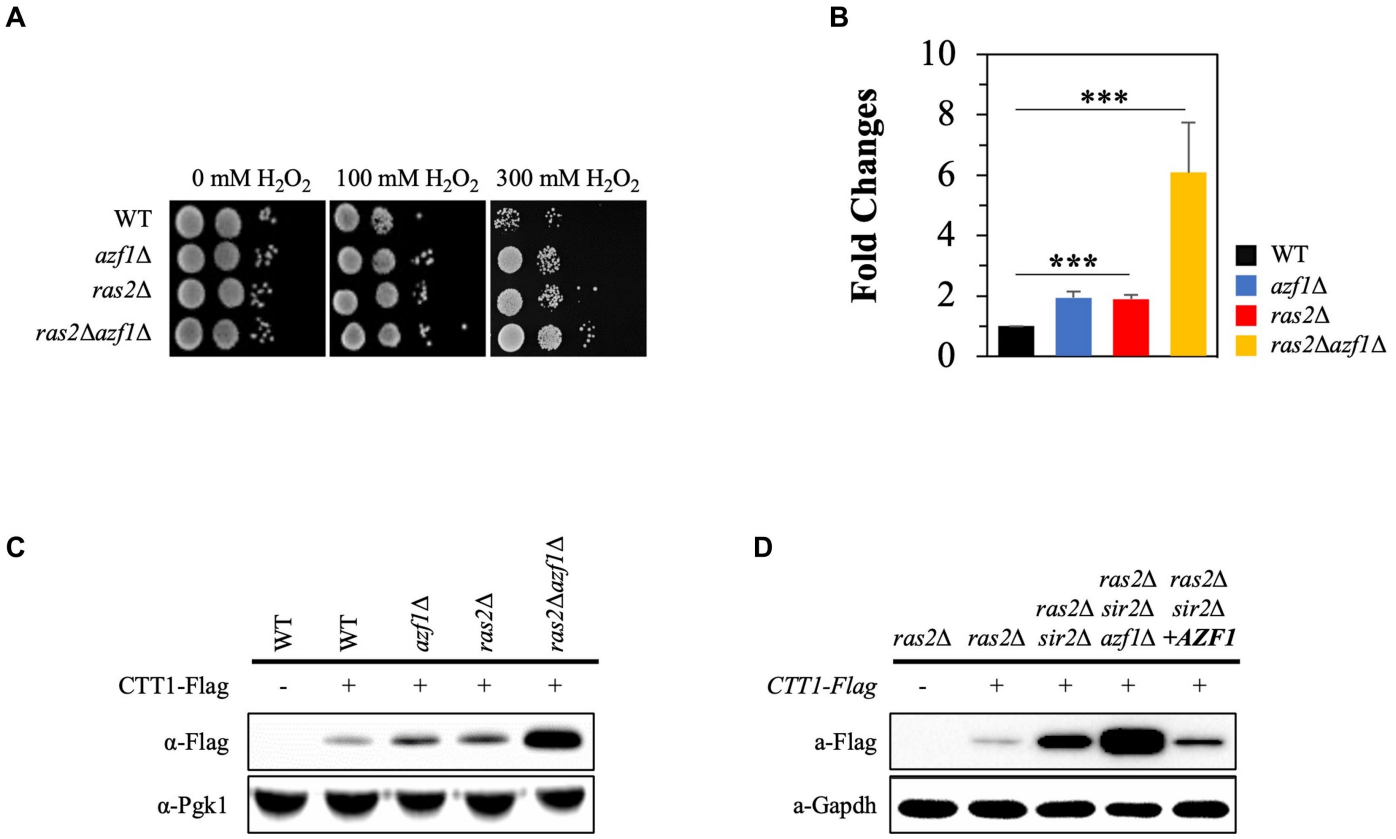
The Azf1 transcription factor represses *CTT1* expression. **(A)** H_2_O_2_ resistance was assessed in the wild-type, *azf1∆*, *ras2*Δ, and *ras2*Δ*azf1* cells in the post-diauxic phase. **(B)** The mRNA levels of *CTT1* were measured by qRT-PCR. The data represent the average of at least three independent experiments (±SD), and *p*-values were calculated using a *t*-test (^***^p < 0.005). **(C)** Ctt1 protein levels in the wild-type, *azf1∆*, *ras2*Δ, and *ras2*Δ*azf1* strains in the post-diauxic phase were measured by western blot. Pgk1 was used as a loading control. **(D)** Azf1 overexpression significantly reduced *CTT1* expression in the *ras2*Δ*sir2*Δ strain. Ctt1 protein levels in *ras2*Δ, *ras2*Δ*sir2*Δ, *ras2*Δ*sir2*Δ*azf1*Δ, *ras2*Δ*sir2*Δ*AZF1*(o/e) strains in the post-diauxic phase were measured by western blot. GAPDH was used as a loading control.

We conducted an in-silico analysis of the *CTT1* promoter region (~1.0 kb) to identify potential transcription factor binding sites. The results revealed numerous binding sites for a range of transcription factors, including Azf1, Cst6, Asg1, and Atf1, as well as well-known ones such as Mns2/4, Skn7, Yap1, and Hsf1 (Lee et al., 1999; Hasan et al., 2002; He and Fassler, 2005) (Supplementary Figure S3). Among these, we paid particular attention to Azf1 because the deletion of *AZF1* compromised cell wall integrity (Slattery et al., 2006), which inhibits PKA signaling (Garcia et al., 2017). Additionally, it was reported that the deletion of *AZF1* further enhanced the increased chronological lifespan of *ras2*∆ (Choi et al., 2013). In this study, we assessed the effect of *AZF1* deletion on H_2_O_2_ resistance in both the wild-type and *ras2*∆ strains. Interestingly, the *azf1*∆ strain showed increased resistance to H_2_O_2_ stress compared to the wild-type strain, and the *ras2*∆*azf1*∆ strain exhibited higher resistance to H_2_O_2_ than the *ras2*∆ strain (Figure 6A). Moreover, the observed H_2_O_2_ resistance phenotypes of the strains corresponded to the levels of the Ctt1 mRNA and protein (Figures 6B,C). Next, we examined the effect of *AZF1* deletion or overexpression on *CTT1* expression in the *ras2*∆*sir2*∆ strain. Deletion of *AZF1* further increased *CTT1* expression, which is already being expressed at a high level in the *ras2*∆*sir2*∆ strain, and overexpression of *AZF1* significantly reduced *CTT1* expression in the *ras2*∆*sir2*∆ strain (Figure 6D). These findings imply that the Azf1 transcription factor may function as a repressor inhibiting the activity of the *CTT1* promoter.

### Sir2 is responsible for maintaining the amount of Azf1

**Figure 7:**
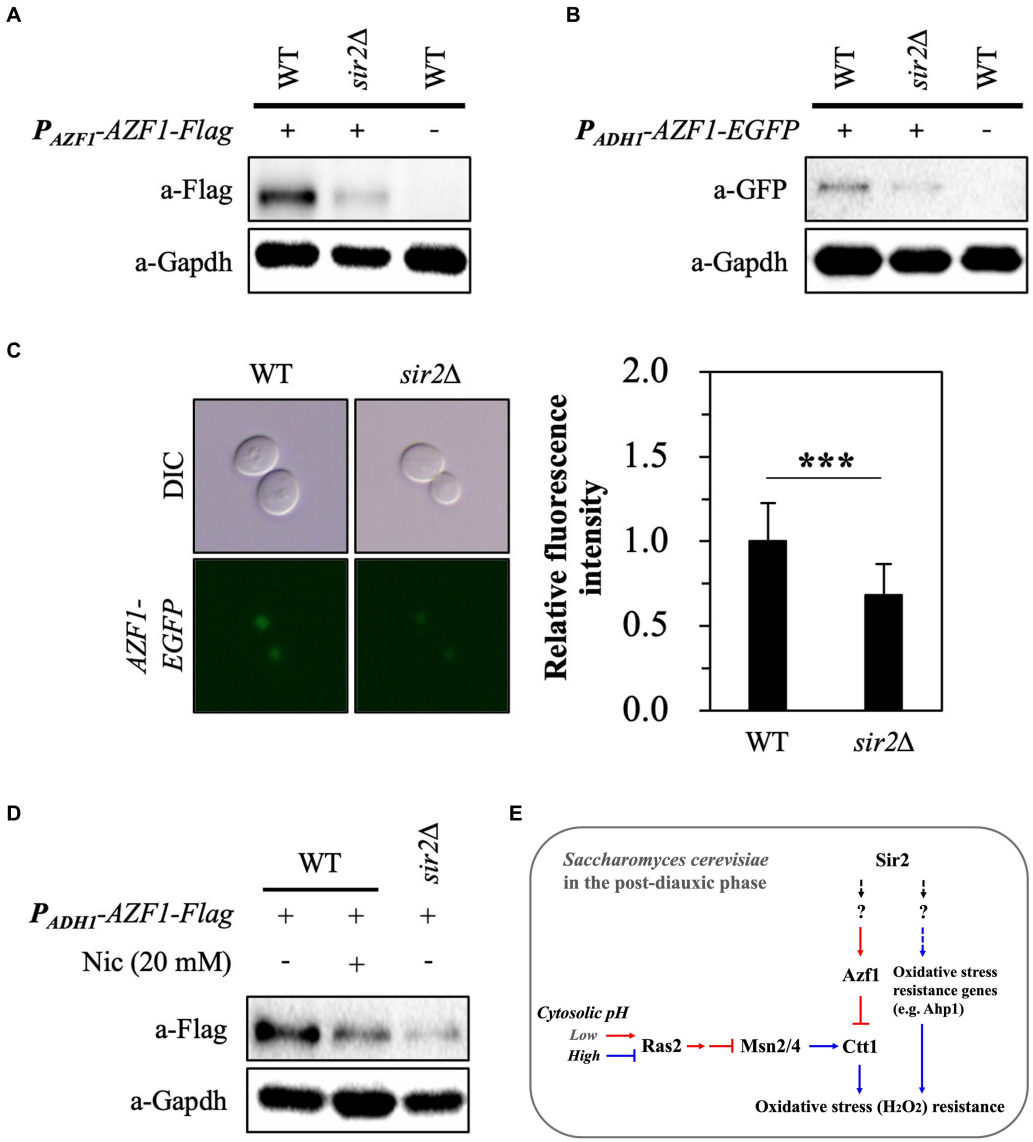
*SIR2* deletion reduces Azf1 protein level. **(A)** The expression level of flag-tagged Azf1 driven by the *AZF1* promoter was analyzed in the wild-type and *sir2∆* cells. **(B)** The expression level of EFGP-tagged Azf1 driven by the *ADH1* promoter was analyzed in the wild-type and *sir2∆* cells. GAPDH was used as a loading control. **(C)** Fluorescence images of the wild-type and *sir2∆* strains expressing *AZF1-EGFP* driven by the *ADH1* promoter. Fluorescence intensity was quantified using ImageJ software. *p*-value was calculated using a *t*-test (^***^p < 0.005). **(D)** Treatment with the Sir2 inhibitor nicotinamide (20 mM) decreased the expression level of Flag-tagged Azf1. **(E)** A schematic diagram illustrating how the absence of Sir2 can result in different levels of oxidative stress resistance during the post-diauxic phase. The red color represents the repression of *CTT1* expression, while blue signifies increased expression of *CTT1* or oxidative stress resistance genes.

To further investigate the association between Azf1 and Sir2 in regulating *CTT1* expression, we examined the levels of Azf1 in the wild-type and *sir2*∆ stains. Because the transcription level of *AZF1* was similar in the wild-type and *sir2*∆ strains (data not shown), we analyzed the amount of Azf1 protein in these strains. Surprisingly, the flag-tagged Azf1 protein level was significantly lower in the *sir2*∆ mutant (Figure 7A). To confirm this observation, we measured the amount of the Azf1-EGFP fusion protein expressed under the *ADH1* promoter and its presence in the nucleus (Figures 7B,C). Additionally, treatment with nicotinamide, a Sir2 inhibitor, also led to a reduction in Azf1 protein levels (Figure 7D). These findings suggest that the interaction between Azf1 and Sir2 may be specific and play a role in regulating the Azf1 protein level.

## Discussion

The regulatory function of Sir2 in cellular metabolism and genomic stability is conserved across diverse organisms (Smith et al., 2007; Schwer and Verdin, 2008). In this study, we found that the deletion of the *SIR2* gene resulted in a differential response of *S. cerevisiae* to oxidative stress, with variations dependent upon the presence of Ras2 during the post-diauxic phase. In the presence of Ras2, the diminished expression of oxidative stress resistance genes in the *sir2*Δ mutant led to an increased sensitivity to oxidative stress. However, in the absence of Ras2, the substantial upregulation of *CTT1* in the *sir2*Δ mutant conferred a strong resistance to oxidative stress on yeast cells in the post-diauxic phase. Our data suggest that the transcription factor Azf1 may act as a repressor in regulating *CTT1* expression, and its abundance appears to decrease in the *sir2*Δ mutant. These findings underscore the significance of metabolic states and environmental conditions in shaping stress response dynamics and show the intricate interplay between Sir2 and other phase-dependent regulatory factors.

During the exponential growth phase, the *sir2*Δ strain exhibited increased resistance to H_2_O_2_ compared to the wild-type in the complex YPD medium and synthetic SDC medium. However, during the post-diauxic phase, the *sir2*Δ strain showed decreased resistance to H_2_O_2_ compared to the wild-type in the YPD medium but not in the SDC medium (Figure 1). The differences in nutrient composition and concentrations between YPD and SDC media could result in alterations in cellular processes, potentially affecting how Sir2 regulates the oxidative stress response (Wierman and Smith, 2014). We analyzed the transcriptomes of the wild-type and *sir2*Δ cells in the exponential or post-diauxic phase. In cells in the exponential phase, when the absence of Sir2 increased resistance to oxidative stress, none of the 47 genes with increased expression were found to be related to oxidative stress. Moreover, the GO analysis of the 130 genes exhibiting decreased expression did not show any characteristics associated with oxidative stress resistance (Supplementary Figure S4). Transcriptome analysis of cells in the post-diauxic phase revealed that several genes associated with oxidative stress resistance, such as *AHP1*, *GCY1*, and *GRX1*, were among the 532 genes downregulated in the *sir2*Δ strain. This change in the *sir2*Δ strain during the post-diauxic phase appears to contribute to the decreased H_2_O_2_ resistance. However, the downregulation of a significant number of genes in the *sir2*Δ mutant does not easily align with the gene-silencing function of Sir2 (Imai et al., 2000; Wierman and Smith, 2014). Further research is needed to elucidate the molecular mechanisms underlying this downregulation in the *sir2*Δ mutant.

The cytosolic pH (pHc) plays a crucial role in oxidative stress resistance during the post-diauxic phase (Figure 3). The relationship between Ras2 activity and pHc suggests that pHc is not simply a passive outcome but an active player in the cellular response, as suggested previously (Orij et al., 2011, 2012). During the post-diauxic phase in the YPD medium, Ras2 activity may be necessary for yeast cells to grow on ethanol before entering the stationary phase. In this context, stress resistance genes such as *MSN2/4* are not expressed due to the active Ras2-cAMP/PKA signaling. When we either increased the cytosolic pH to inhibit Ras2 activity or deleted *RAS2*, Msn2/4 transcription factors were activated, leading to upregulated *CTT1* expression. However, deletion of *MSN2/4* in both *ras2*Δ and *ras2*Δ*sir2*Δ mutants indicated that, while Msn2/4 play a significant role in *CTT1* expression (Supplementary Figure S3), they do not fully account for the dramatic increase of *CTT1* expression in the absence of Sir2. This finding implies the existence of additional regulatory elements or pathways influencing *CTT1* expression. A previous study identified a region within the *CTT1* promoter associated with the negative regulation of *CTT1* expression (Belazzi et al., 1991), raising the possibility that a repressor may be involved in this regulation.

One of the notable findings of this study is the role of Azf1 in the intricate interactions among Ras2, Sir2, and *CTT1* expression. Our discovery that the *CTT1* promoter region contains Azf1 targeting sequences, specifically AAAAGAAA (A_4_GA_3_), from positions-779 to-791, when coupled with a previous study showing that deletion of *AZF1* significantly enhanced the chronological lifespan (CLS) of *ras2* mutants, led us to hypothesize that Azf1 could be closely linked to *CTT1* expression. Azf1 transcription factor belongs to the C2H2 zinc finger class and has a largely distinct set of target genes during growth in fermentable and non-fermentable carbon sources (Stein et al., 1998; Newcomb et al., 2002; Slattery et al., 2006). In the presence of glucose, Azf1 activates the transcription of genes involved in growth and carbon metabolism, such as *SIP4* and *VID24*. Conversely, during growth in non-fermentable carbon sources, the deletion of *AZF1* was shown to increase the transcription of genes related to cell wall biogenesis and organization, including *GAS1* and *GAS3* (Slattery et al., 2006). Intriguingly, our study suggests that Azf1 may function as a repressor in the context of *CTT1* expression (Figure 6), underscoring the multifaceted roles of Azf1 as both an activator and a repressor in cellular growth and stress responses.

The differential protein levels of Azf1 between the wild-type and *sir2*Δ strains in the absence of transcriptional differences suggest a post-transcriptional regulatory role for Sir2 in regulating Azf1 (Figure 7). This regulation could involve mechanisms related to protein stabilization or the regulation of degradation pathways. Indeed, previous studies have indicated that Sir2 is associated with the stability of non-histone proteins (Lin et al., 2009; Howie et al., 2019). A critical challenge for future research will be to unravel the nature of this relationship—specifically, whether Sir2 interacts directly with Azf1 or modulates its levels through intermediary proteins or pathways.

In conclusion, this study has advanced the understanding of Sir2’s complex roles in the oxidative stress response and provided valuable insights into the intricate regulatory interactions among Sir2, Azf1, and *CTT1* expression in yeast. Future research could aim to delineate the molecular mechanisms underpinning these observations, including the role of physiological state and environmental factors like nutrient availability in modulating Sir2’s functions. Additional studies are essential for elucidating the precise mechanisms behind these interactions and understanding their implications for cellular physiology and adaptation.

## Data availability statement

The datasets presented in this study can be found in online repositories. The names of the repository/repositories and accession number(s) can be found at: https://figshare.com/, doi.org/10.6084/m9.figshare.24055566.v1.

## Author contributions

YHK: Investigation, Writing – original draft. J-IR: Investigation, Validation, Visualization, Writing – review & editing. MD: Investigation, Writing – review & editing. JJ: Investigation, Writing – review & editing. J-YK: Conceptualization, Funding acquisition, Resources, Supervision, Writing – original draft, Writing – review & editing.
