# Corrigendum: The intricate role of Sir2 in oxidative stress response during the post-diauxic phase in *Saccharomyces cerevisiae*

**DOI:** 10.3389/fmicb.2023.1357693

**Published:** 2024-01-08

**Authors:** Yeong Hyeock Kim, Ji-In Ryu, Mayur Nimbadas Devare, Juhye Jung, Jeong-Yoon Kim

**Affiliations:** Department of Microbiology and Molecular Biology, College of Bioscience and Biotechnology, Chungnam National University, Daejeon, Republic of Korea

**Keywords:** *Saccharomyces cerevisiae*, Sir2, oxidative stress, Ras2, cytosolic pH, Azf1

In the published article, there was an error in Figure 4C and its caption. The constitutively active form of *RAS2* was incorrectly labelled as “*RAS*2^*Q*19*V*^”. The correct expression is “*RAS*2^*G*19*V*^”.

The corrected Figure 4C and its revised caption appear below.

**Figure 4:**
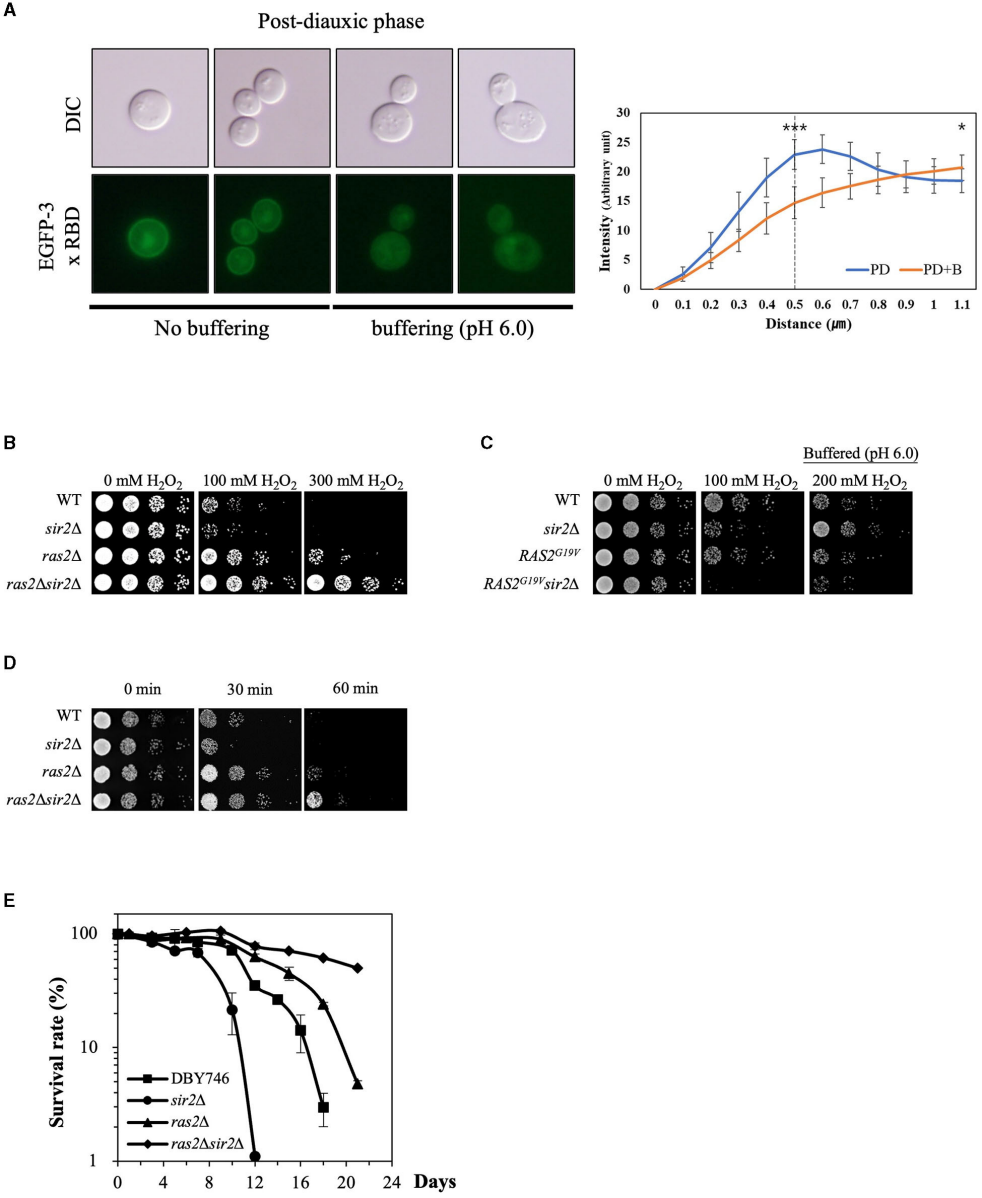
Ras2 activity is involved in the altered effect of *SIR2* deletion on H_2_O_2_ resistance during post-diauxic growth phases. **(A)** (Left panel) Ras2 activity was analyzed using EGFP-3x RBD in the wild-type strains treated with or without buffer (pH 6.0). (Right panel) The fluorescence intensity was assessed using ImageJ and expressed in arbitrary units (*n* = 10 cells for each strain). The distance is measured starting from a point outside the cell and traversing through the cell membrane. The dotted line at 0.5 μm serves as indicator of the approximate boundary between the interior and exterior of the membrane. *p*-values were calculated using a *t*-test (^*^p < 0.05 and ^***^p < 0.005). **(B)** H_2_O_2_ resistance was evaluated in the wild-type, *sir2*Δ, *ras2*Δ, and *ras2*Δ*sir2*Δ cells during the post-diauxic phase. **(C)** H_2_O_2_ resistance was tested in the wild-type, *sir2*Δ, *RAS2*^*G*19*V*^, and *RAS2*^*G*19*V*^*sir2*Δ strains during the post-diauxic phase. Note that *RAS2*^*G*19*V*^ is a constitutively active form of *RAS2*. **(D)** Heat stress resistance was evaluated in the wild-type, *sir2*Δ, *ras2*Δ, and *ras2*Δ*sir2*Δ cells during the post-diauxic phase. **(E)** The chronological lifespan of the wild-type, *sir2*Δ, *ras2*Δ, and *ras2*Δ*sir2*Δ strains grown in YPD medium was monitored by counting colony-forming units every 2 or 3 days. Experiments were repeated three times. Error bars indicate the mean ± SD.

In the published article, there was an error in Figure 5B as published. Three asterisks (^*^) were used to denote *p* < 0.05. This has been corrected to one asterisk. There was an error in the caption for Figure 5. The types “*ctt11*Δ” and “*ras2*Δ*ctt11*Δ” were incorrectly used. The correct expressions are “*ctt1*Δ” “*ras2*Δ*ctt1*Δ”.

The corrected Figure 5B and its caption appear below.

**Figure 5:**
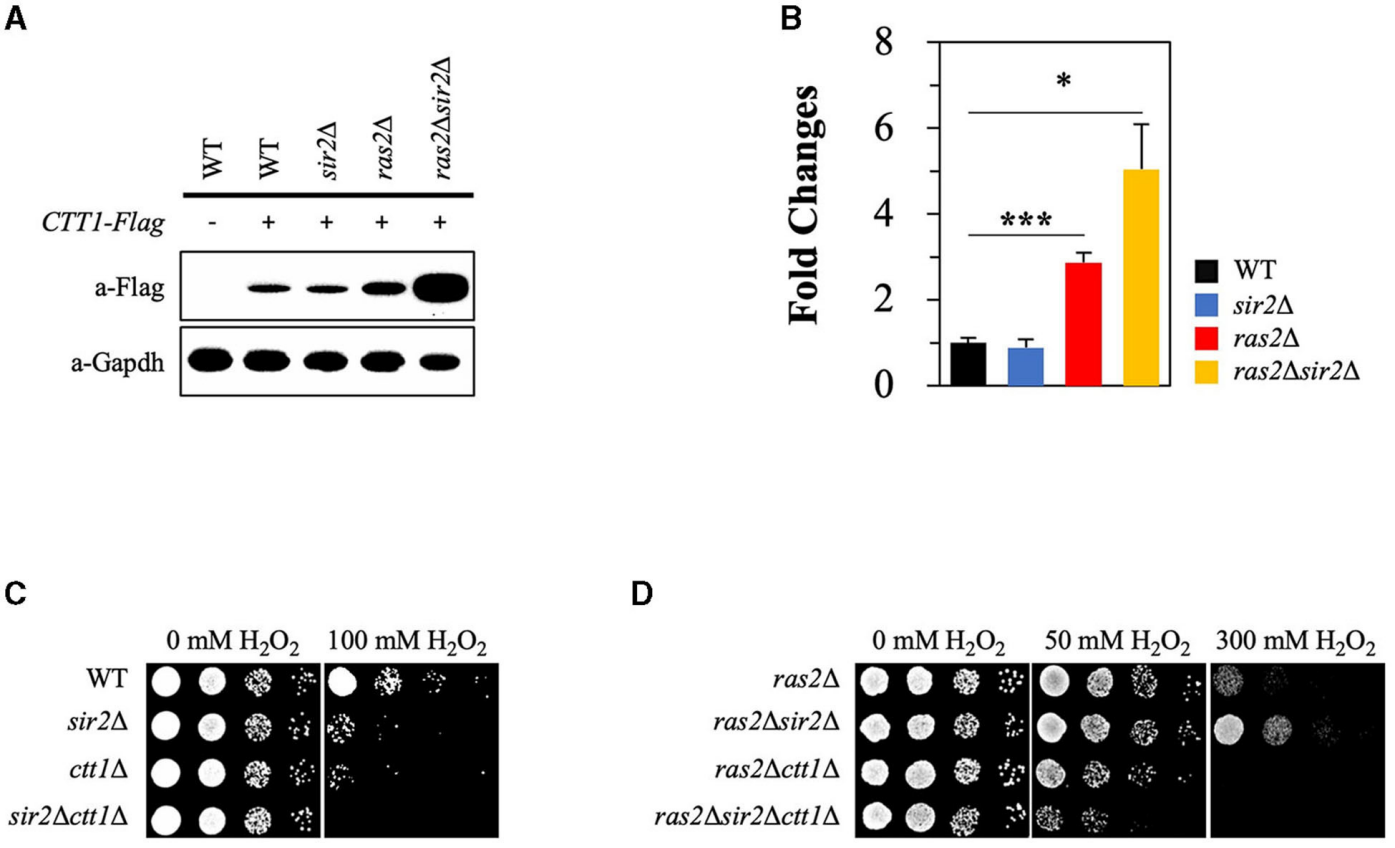
Sir2 affects the expression of *CTT1* in the absence of Ras2 but not in its presence. **(A)** Ctt1 protein levels in the wild-type, *sir2*Δ, *ras2*Δ, and *ras2*Δ*sir2*Δ strains were measured by western blot. GAPDH was used as a loading control. **(B)** qRT-PCR was performed to assess *CTT1* mRNA levels in the wild-type, *sir2*Δ, *ras2*Δ, and *ras2*Δ*sir2*Δ. The data represent the average of at least three independent experiments (±SD), and *p*-values were calculated using a *t*-test (^*^p < 0.05 and ^***^p < 0.005). **(C)** H_2_O_2_ resistance was tested in the wild-type, *sir2*Δ, *ctt1*Δ, and *sir2*Δ*ctt1*Δ strains. **(D)** H_2_O_2_ resistance was also tested in the *ras2*Δ, *ras2*Δ*sir2*Δ, *ras2*Δ*ctt1*Δ, and *ras2*Δ*sir2*Δ*ctt1*Δ strains during the post-diauxic phase.

In the published article, there was an error in **Results**, “Ras2 is responsible for different responses to H_2_O_2_ stress during the post-diauxic phase”, paragraph 2, lines 8–10.

This sentence previously stated:

“In addition, we found that the expression of a constitutively active form of *RAS2* (*RAS2*^*Q*19*V*^) increased the sensitivity of the *sir2*Δ cells to H_2_O_2_ stress (Figure 4C).”

The corrected sentence appears below:

“In addition, we found that the expression of a constitutively active form of *RAS2* (*RAS2*^*G*19*V*^) increased the sensitivity of the *sir2*Δ cells to H_2_O_2_ stress (Figure 4C).”

The authors apologize for these errors and state that they do not change the scientific conclusions of the article in any way. The original article has been updated.

