# Supplementary material for: The intricate role of Sir2 in oxidative stress response during the post-diauxic phase in Saccharomyces cerevisiae
Source: Front Microbiol. 2023 Nov 9;14:1285559. doi: 10.3389/fmicb.2023.1285559 (PMC10666771; doi:10.3389/fmicb.2023.1285559)
Supplement: Supplementary file 1 [file Presentation_1.PPTX]

## Slide 1
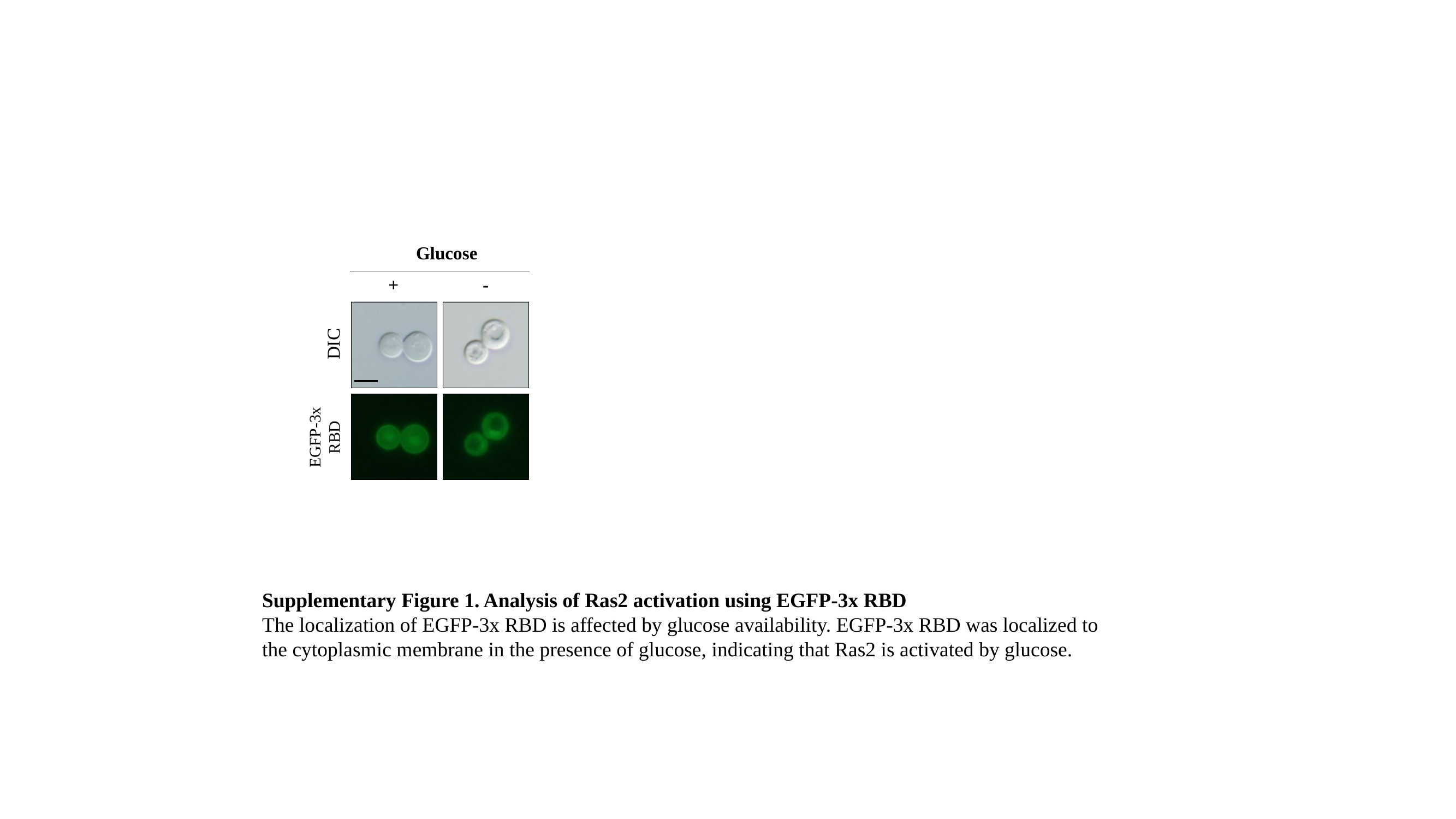

Glucose
+
-
DIC
EGFP-3x RBD
Supplementary Figure 1. Analysis of Ras2 activation using EGFP-3x RBD
The localization of EGFP-3x RBD is affected by glucose availability. EGFP-3x RBD was localized to the cytoplasmic membrane in the presence of glucose, indicating that Ras2 is activated by glucose.

## Slide 2
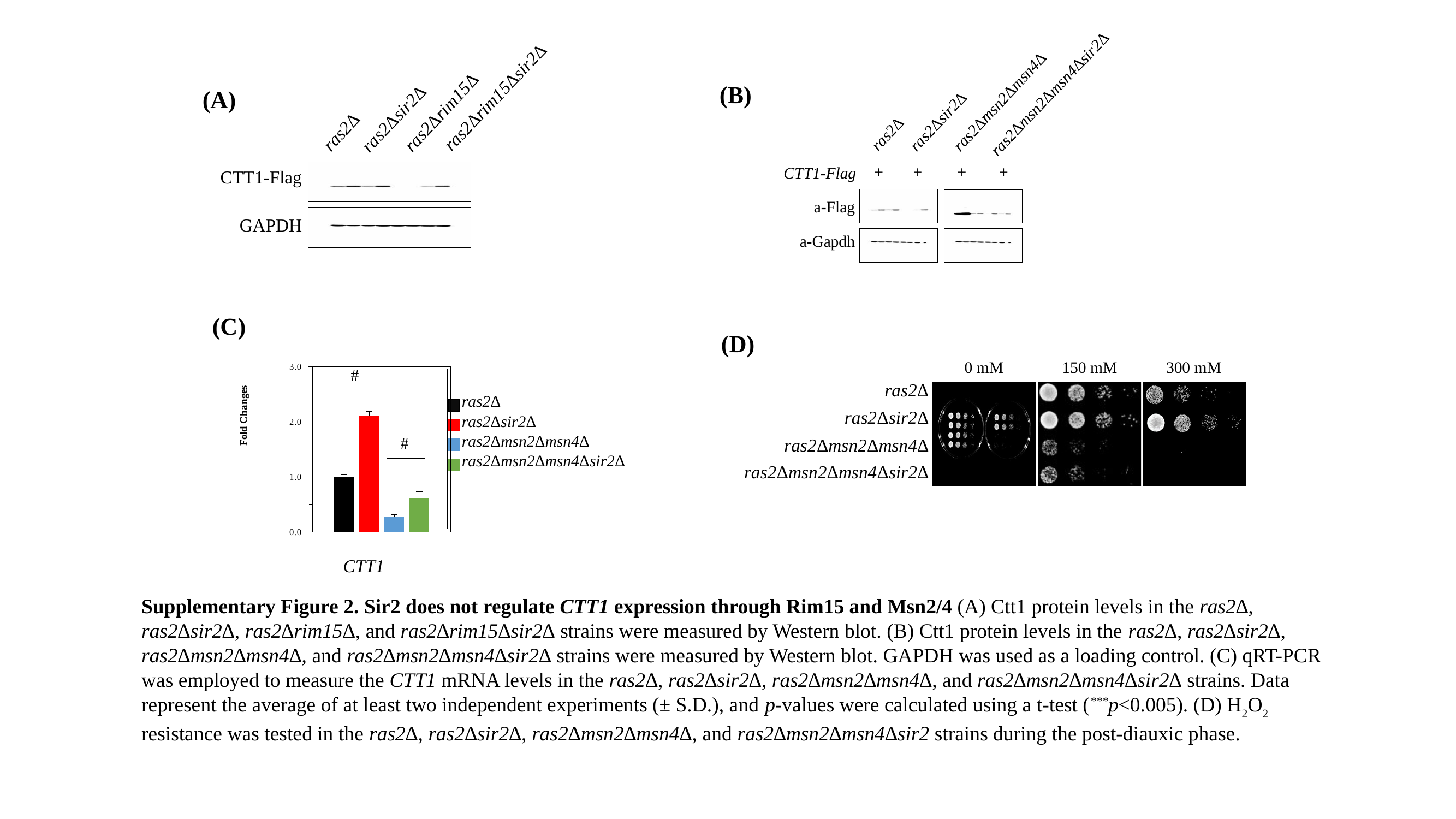

ras2Δmsn2Δmsn4Δsir2Δ
ras2Δmsn2Δmsn4Δ
ras2Δsir2Δ
ras2Δ
+
+
+
+
CTT1-Flag
a-Flag
a-Gapdh
(B)
ras2Δrim15Δsir2Δ
ras2Δrim15Δ
ras2Δsir2Δ
ras2Δ
CTT1-Flag
GAPDH
(A)
(C)
### Chart
| Category | ras2Δ | ras2Δsir2Δ | ras2Δmsn2Δmsn4Δ | ras2Δmsn2Δmsn4Δsir2Δ |
|---|---|---|---|---|
| CTT1 | 1.0 | 2.1068400938861505 | 0.2711447240327352 | 0.617139713165487 |#
#
ras2Δ
ras2Δsir2Δ
ras2Δmsn2Δmsn4Δ
ras2Δmsn2Δmsn4Δsir2Δ
CTT1
(D)
300 mM
0 mM
150 mM
ras2Δ
ras2Δsir2Δ
ras2Δmsn2Δmsn4Δ
ras2Δmsn2Δmsn4Δsir2Δ
Supplementary Figure 2. Sir2 does not regulate CTT1 expression through Rim15 and Msn2/4 (A) Ctt1 protein levels in the ras2∆, ras2∆sir2∆, ras2∆rim15∆, and ras2∆rim15∆sir2∆ strains were measured by Western blot. (B) Ctt1 protein levels in the ras2∆, ras2∆sir2∆, ras2∆msn2∆msn4∆, and ras2∆msn2∆msn4∆sir2∆ strains were measured by Western blot. GAPDH was used as a loading control. (C) qRT-PCR was employed to measure the CTT1 mRNA levels in the ras2∆, ras2∆sir2∆, ras2∆msn2∆msn4∆, and ras2∆msn2∆msn4∆sir2∆ strains. Data represent the average of at least two independent experiments (± S.D.), and p-values were calculated using a t-test (***p<0.005). (D) H2O2 resistance was tested in the ras2∆, ras2∆sir2∆, ras2∆msn2∆msn4∆, and ras2∆msn2∆msn4∆sir2 strains during the post-diauxic phase.

## Slide 3
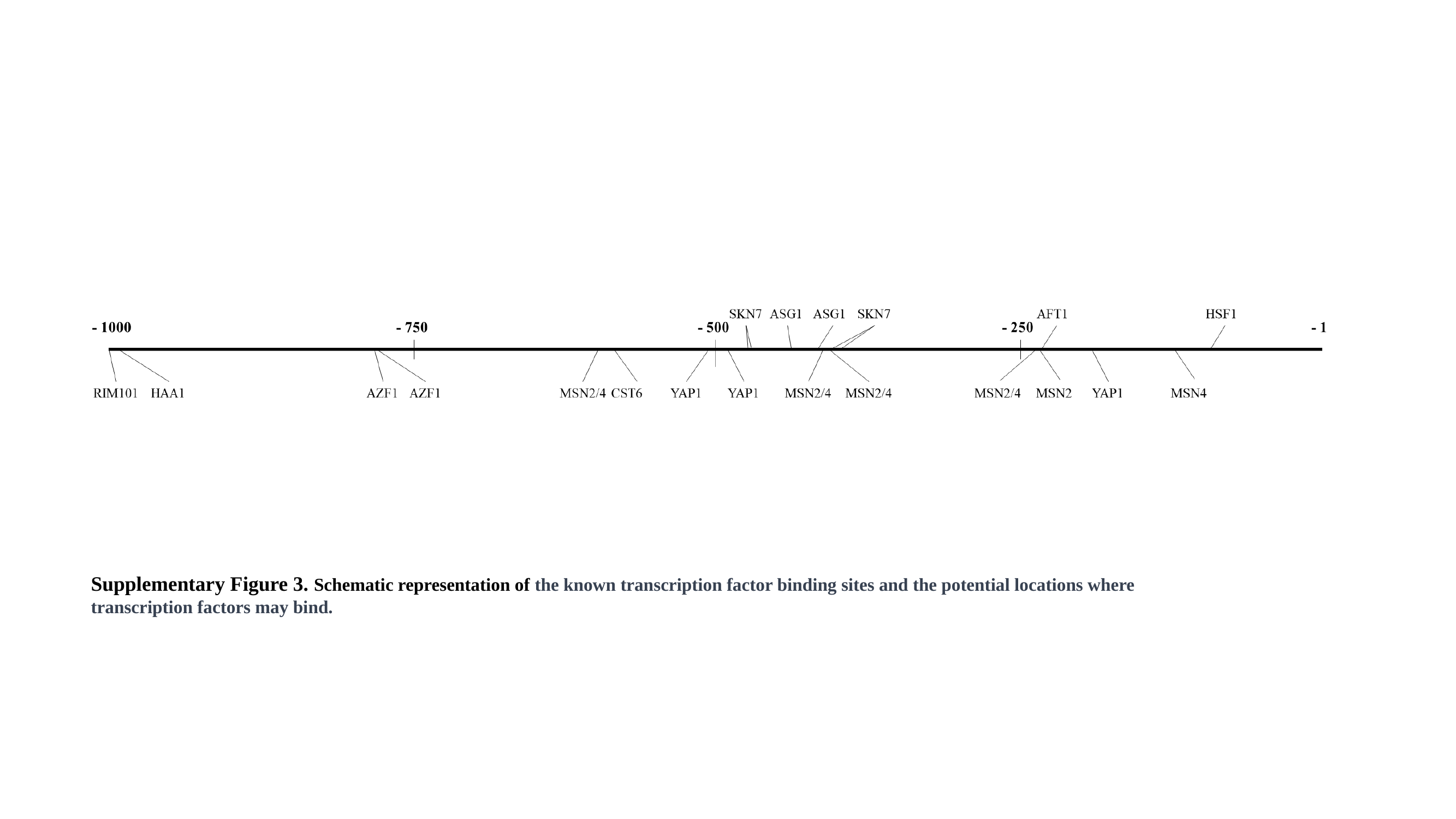

Supplementary Figure 3. Schematic representation of the known transcription factor binding sites and the potential locations where transcription factors may bind.

## Slide 4
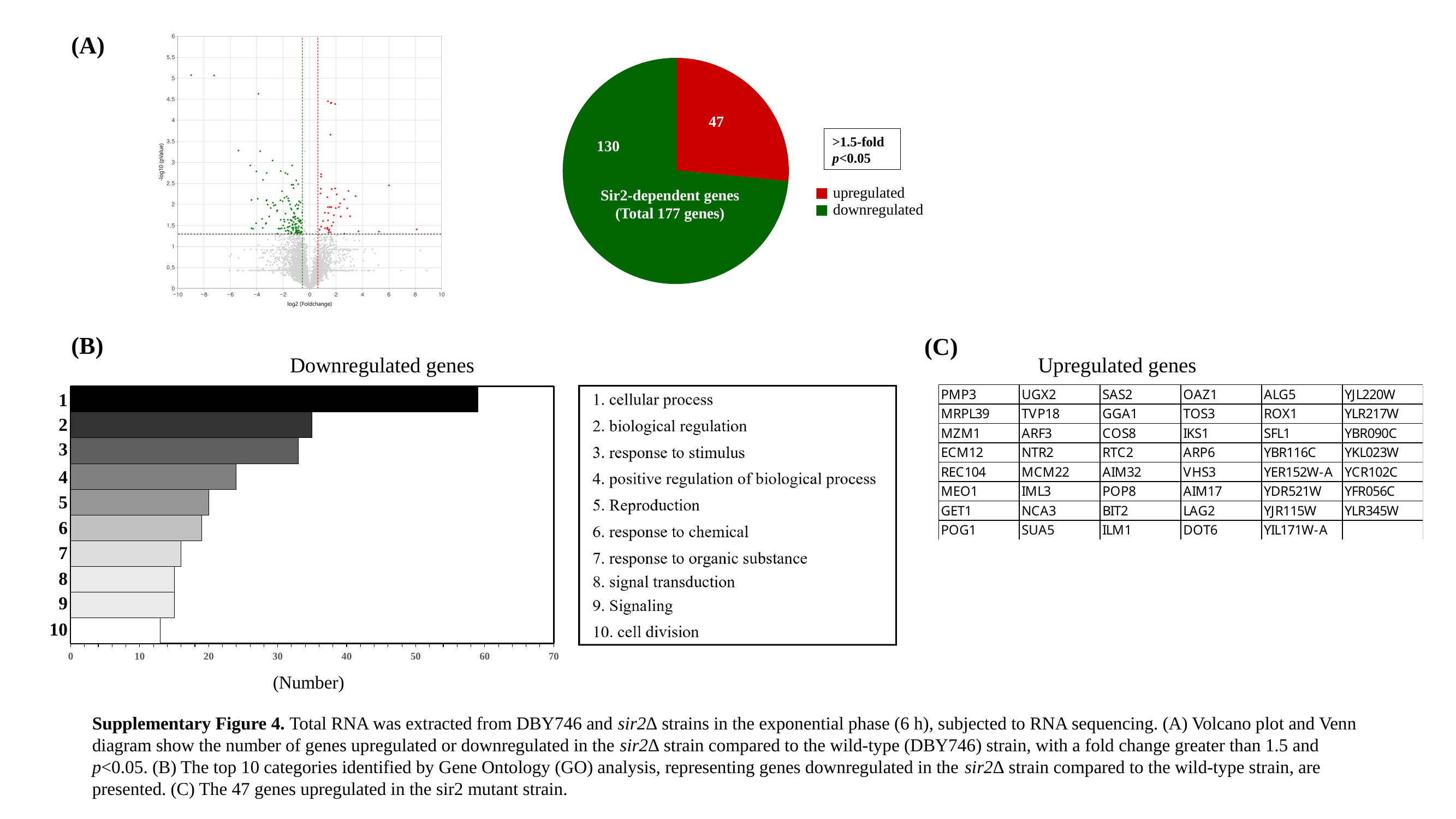

(A)
### Chart
| Category | |
|---|---|47
130
Sir2-dependent genes
(Total 177 genes)
>1.5-fold
p<0.05
upregulated
downregulated
(B)
(C)
Downregulated genes
Upregulated genes
### Chart
| Category | |
|---|---|
| 10 | 13.0 |
| 9 | 15.0 |
| 8 | 15.0 |
| 7 | 16.0 |
| 6 | 19.0 |
| 5 | 20.0 |
| 4 | 24.0 |
| 3 | 33.0 |
| 2 | 35.0 |
| 1 | 59.0 |1
2
3
4
5
6
7
8
9
10
(Number)
Supplementary Figure 4. Total RNA was extracted from DBY746 and sir2∆ strains in the exponential phase (6 h), subjected to RNA sequencing. (A) Volcano plot and Venn diagram show the number of genes upregulated or downregulated in the sir2∆ strain compared to the wild-type (DBY746) strain, with a fold change greater than 1.5 and p<0.05. (B) The top 10 categories identified by Gene Ontology (GO) analysis, representing genes downregulated in the sir2∆ strain compared to the wild-type strain, are presented. (C) The 47 genes upregulated in the sir2 mutant strain.
